# Supplementary material for: NMR Lipid Profile of Milk from Alpine Goats with Supplemented Hempseed and Linseed Diets
Source: Molecules. 2020 Mar 25;25(7):1491. doi: 10.3390/molecules25071491 (PMC7181232; doi:10.3390/molecules25071491)
Supplement: Supplementary file 1 [file molecules-25-01491-s001.pdf]

NMR lipid profile of milk from Alpine goats with supplemented hempseed and linseed diets.

Antonella Caterina Boccia *[1], Erica Cusano[1], Paola Scano[2], and Roberto Consonni[1]

[1] CNR, Istituto di Scienze e Tecnologie Chimiche " Giulio Natta"-SCITEC, via A. Corti, 12; 20133 Milano Italy; e-mail: antonella.boccia@scitec.cnr.it (A.C.B.); erica.cusano@cnr.it (E.C.); roberto.consonni@scitec.cnr.it (R.C.)

[2] University of Cagliari, Department of Life and Environmental Sciences, via Ospedale 72, 09124, Cagliari, Italy; e-mail: scano@unica.it (P.S.)

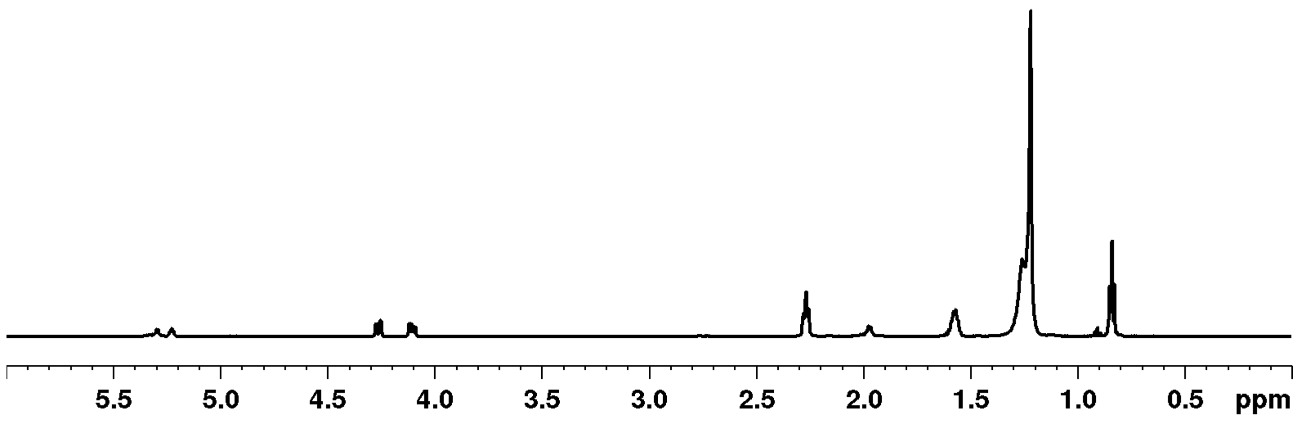

Figure S1. ^{1}H spectrum of goat milk lipid fraction, recorded on a 600 MHz spectrometer, in $CDCl_3$ at 298 K.

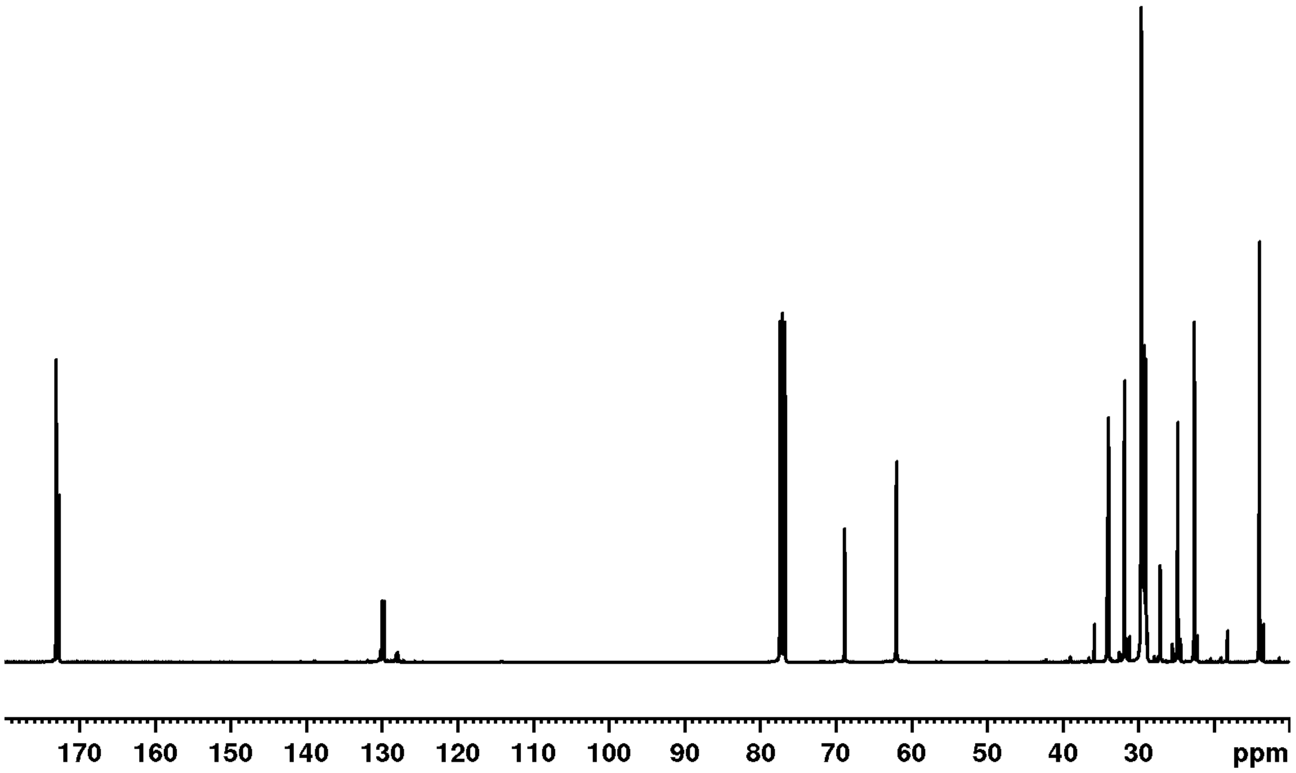

Figure S2. ^{13}C spectrum of goat milk lipid fraction, recorded on a 400 MHz spectrometer, in $CDCl_3$ at 298 K.

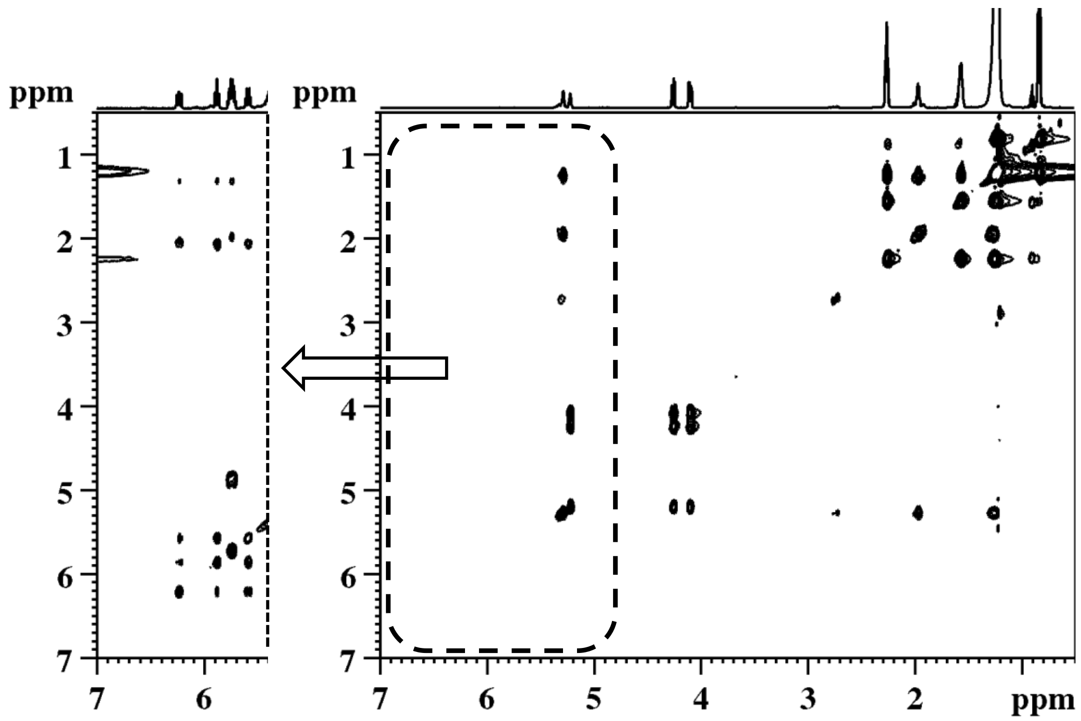

Figure S3. ^{1}H- ^{1}H TOCSY spectrum of goat milk lipid fraction, recorded on a 600 MHz spectrometer, in $CDCl_3$ at 298 K.

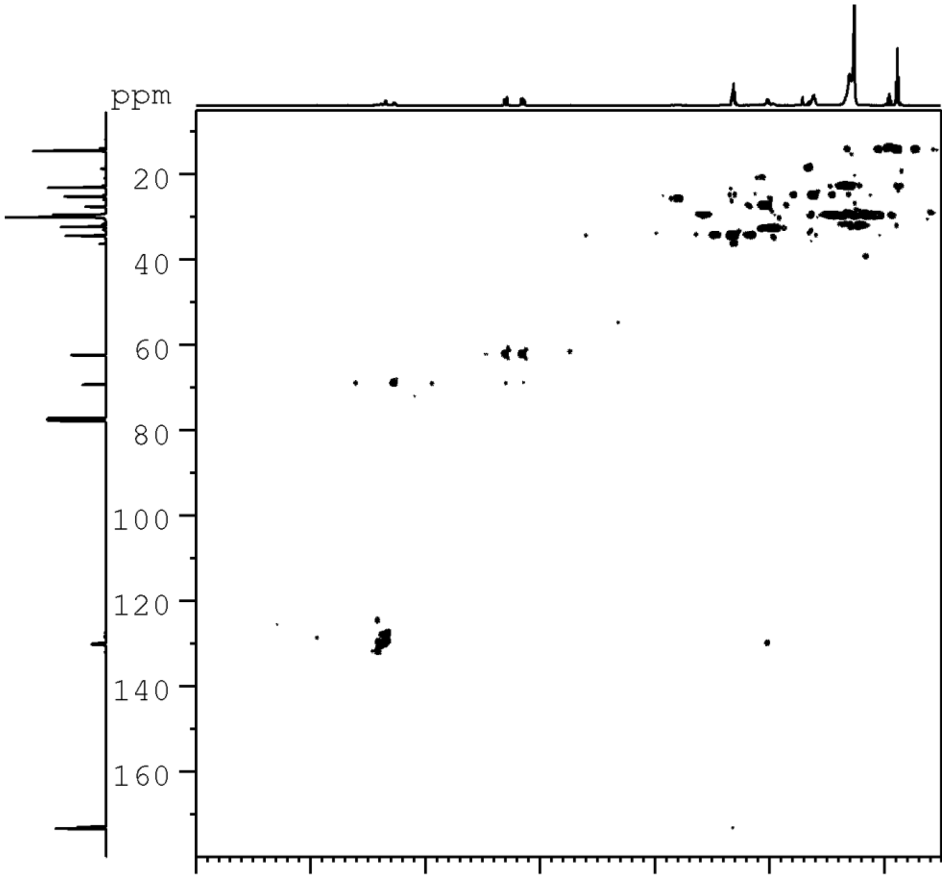

Figure S4. ^{1}H- ^{13}C HSQC full spectrum of goat milk lipid fraction, recorded on a 600 MHz spectrometer, in $CDCl_3$ at 298 K.

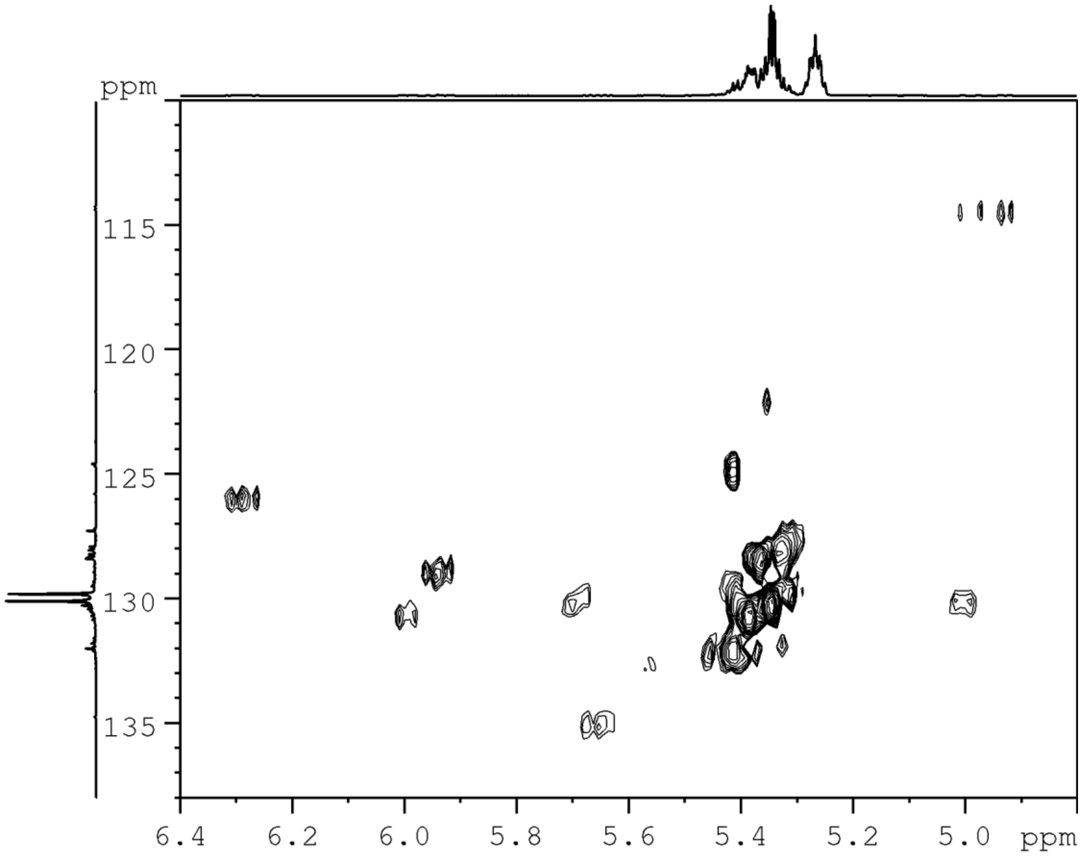

Figure S5. Expandend region of ^{1}H- ^{13}C HSQC spectrum of goat milk lipid fraction, recorded on a 600 MHz spectrometer, in $CDCl_3$ at 298 K.

Table 1S. Composition of the administered control (C), linseed (L) or hemp (H) diets (% on dry matter).[ref13]

Ingredients	C	L	H
Meadow hay second cut[1]	23.9	23.9	23.9
Meadow hay first cut[1]	13.6	13.6	13.6
Alfalfa hay third cut[1]	10.2	10.2	10.2
Corn flakes[1,2]	17.4	12.3	12.3
Corn meal[1]	5.1	5.1	5.1
Soybean hulls[1,2]	12.9	12.9	12.9
Solvent soybean meal[1,2]	6.2	2.6	2.6
Carob pulp[1]	4.4	4.4	4.4
Cane molasses[2]	0.5	0.5	0.5
Salts[2]	1.4	1.4	1.4
Vitamins[2]	0.3	0.3	0.3
Linseeds[2]		8.5	
Hemp seeds[2]			8.5

1) ingredients of the basal ration (86.5%); 2) ingredients of supplement concentrates (13.5%).

13 Cremonesi, P., Conte, G., Severgnini, M., Turri, F., Monni, A., Capra, E., Rapetti, L., Colombni, S., Chessa, S., Battelli, G., Alves, S. P., Mele, M., Castigioni, B. Evaluation of the effects of different diets on microbiome diversity and fatty acid composition of rumen liquor in dairy goat. Animal, **2018**, 12, 9, 1856-1866
